# Erythropoietin in Tumor Immunity: Mechanistic Evidence, Clinical Cautions, and Translational Opportunities

**DOI:** 10.3390/cancers18183011

**Published:** 2026-09-17

**Authors:** Tongsheng Yang, Linxing Zheng, Tian Lan

**Affiliations:** 1 Department of General Surgery, West China Hospital, Sichuan University, Chengdu 610041, China; yangtongsheng@stu.scu.edu.cn (T.Y.);; 2 Liver Transplant Center, West China Hospital, Sichuan University, Chengdu 610041, China; 3 Laboratory of Hepatic AI Translation, Frontiers Science Center for Disease-Related Molecular Network, West China Hospital, Sichuan University, Chengdu 610041, China

**Keywords:** erythropoietin, erythropoietin receptor, tumor immunity, tumor microenvironment, macrophage, dendritic cell, CD71-positive nucleated erythroid cell, immunotherapy

## Abstract

Erythropoietin (EPO) is a hypoxia-responsive glycoprotein hormone that sustains erythroid progenitor survival through the erythropoietin receptor (EPOR). Beyond erythropoiesis, emerging evidence places the EPO/EPOR axis at the interface of tumor adaptation, stromal remodeling, and immune tolerance. Interpretation has been complicated by nonspecific antibodies, low receptor abundance, and failure to distinguish EPOR transcripts or intracellular proteins from ligand-accessible, functional cell-surface receptors. Recent mechanistic studies have established immunoregulatory roles: tumor-derived EPO reprograms EPOR-positive macrophages through nuclear factor erythroid 2-related factor 2 (NRF2)-dependent heme depletion, whereas EPOR signaling in conventional type 1 dendritic cells promotes tolerance and restrains antitumor T-cell priming. EPO-driven expansion of CD71-positive nucleated erythroid cells provides an additional systemic suppressive route. Evidence for direct effects on tumor cell survival, angiogenesis, and treatment resistance is more heterogeneous and must be interpreted in light of receptor validation, model context, and exposure conditions. This critical narrative review introduces operational evidence categories, distinguishes exploratory tissue screening from causal validation, and proposes a scalable experimental framework with minimum, enhanced, and gold-standard levels. Clinically, erythropoiesis-stimulating agents remain supportive care agents, not anticancer therapies; their use is restricted by thromboembolic risk and historical survival concerns. Translational development should prioritize practical biomarker panels and tumor- or cell-selective interventions that preserve erythropoietic safety.

## Simple Summary

Erythropoietin (EPO) is best known as the hormone that stimulates red blood cell production and is used to treat selected forms of anemia. Cancer-related EPO biology is broader and more context-dependent. Recent studies show that tumor-derived EPO can reprogram macrophages and dendritic cells, while stress erythropoiesis can expand immunosuppressive CD71-positive nucleated erythroid cells. By contrast, evidence for direct EPO signaling in malignant cells remains inconsistent because receptor abundance is low, detection methods vary, and receptor transcripts do not necessarily indicate functional cell-surface signaling. This review differs from earlier reviews focused on tumor cells and angiogenesis by organizing the evidence according to the source of EPO, the responding cellular compartment, and the strength of causal evidence. It also separates practical screening methods from definitive functional validation. Clinically, erythropoiesis-stimulating agents remain supportive care agents with established restrictions; they are not anticancer therapies. The main translational opportunity is to identify tumor-localized, immunosuppressive EPO signaling that might be interrupted without compromising erythropoiesis or vascular safety.

## 1. Introduction

**Figure 1:**
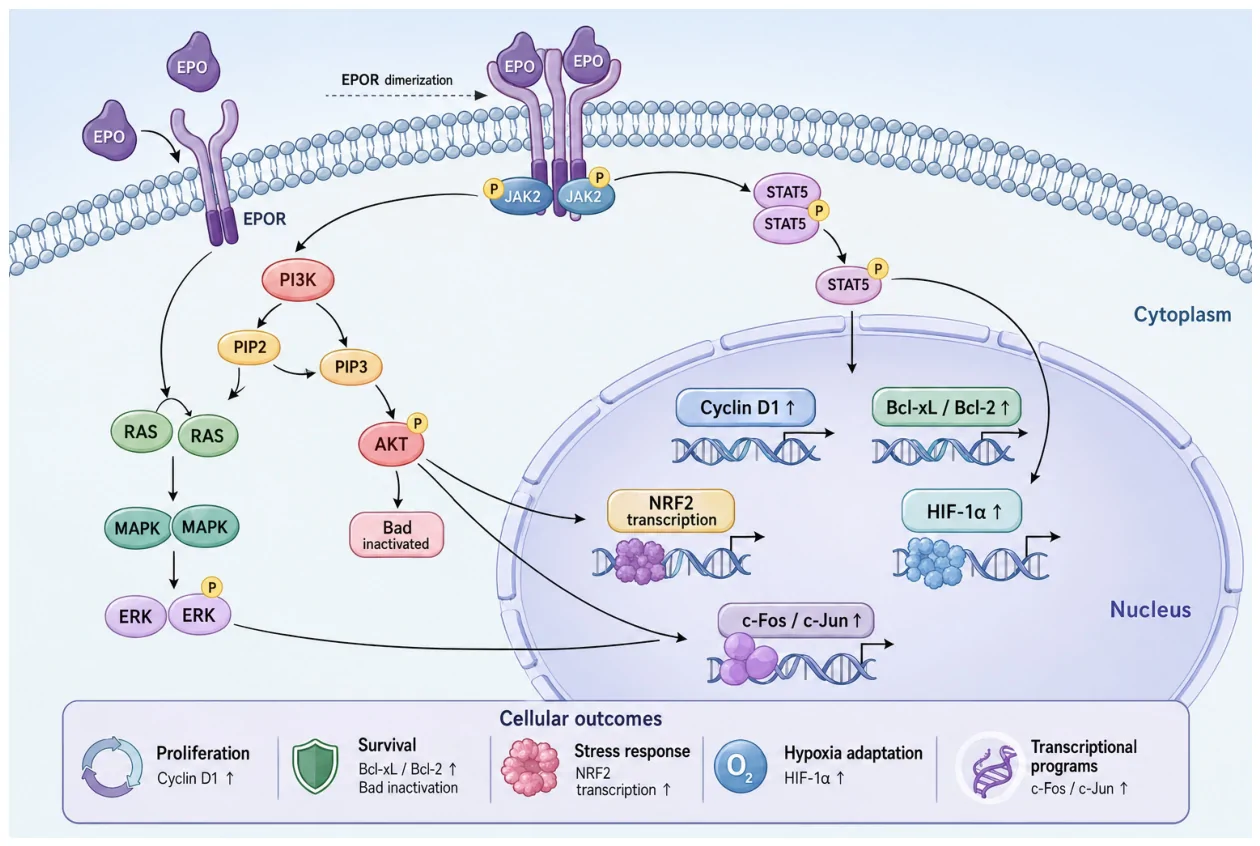
Canonical EPO/EPOR signaling and the evidentiary boundary for extrapolation to cancer. EPO binding changes the geometry of the EPOR complex and activates JAK2 and the STAT5, PI3K/AKT, and RAS/RAF/MEK/ERK branches, which converge on survival and proliferative transcriptional programs. The pathway is well established in erythroid progenitors. In tumor and non-erythroid cells, ligand-accessible receptor and functional signaling should be evaluated with validated, context-appropriate methods rather than inferred from transcript abundance or unvalidated immunostaining. AI assistance was used during the initial visual design of this conceptual schematic. The current figure was drawn by the authors using graphical software and was scientifically verified by the authors; it contains no primary experimental data. Abbreviations: EPO, erythropoietin; EPOR, erythropoietin receptor; HIF-1α, hypoxia-inducible factor 1-alpha; JAK2, Janus kinase 2; MAPK, mitogen-activated protein kinase; NRF2, nuclear factor erythroid 2-related factor 2; PI3K, phosphoinositide 3-kinase; STAT5, signal transducer and activator of transcription 5. Arrows indicate the direction of signal transduction.

Erythropoietin (EPO) is the principal endocrine regulator of red blood cell production. In adults, renal interstitial fibroblast-like cells increase EPO synthesis when oxygen delivery falls, and circulating EPO preserves erythroid progenitors that would otherwise undergo apoptosis. This physiologic response is mediated by the erythropoietin receptor (EPOR), a type I cytokine receptor whose canonical signaling architecture includes Janus kinase 2 (JAK2), signal transducer and activator of transcription 5 (STAT5), phosphoinositide 3-kinase (PI3K)/AKT, and RAS/mitogen-activated protein kinase (MAPK) pathways (Figure 1) [1,2,3]. Recombinant erythropoiesis-stimulating agents (ESAs) transformed the management of anemia, but their use in oncology raised questions that remain clinically relevant: Can malignant or stromal cells respond directly to EPO? Can EPO reshape the tumor immune microenvironment? Can the pathway be manipulated without compromising erythropoiesis or vascular safety?

Earlier reviews primarily examined non-hematopoietic EPOR expression, tumor cell proliferation and survival, angiogenesis, and the clinical safety of ESAs [4,5,6,7]. Those analyses were essential, particularly in exposing the limitations of early EPOR antibodies. However, the field has since shifted from a tumor cell-centered model to a multicellular immune framework. Recent causal studies show that tumor-derived EPO can act on EPOR-positive macrophages to generate a non-inflamed microenvironment and that EPOR signaling in conventional type 1 dendritic cells (cDC1s) promotes immune tolerance [8,9,10]. Tumor-associated stress erythropoiesis also generates immunosuppressive CD71-positive nucleated erythroid cells, and EPO can modify T-cell, B-cell, and myeloid responses in context-dependent settings [11,12,13,14,15,16].

This review has four distinctive features. First, it organizes the literature around a source–responder–effect chain rather than treating “EPO exposure” as a single entity. Second, it separates endogenous circulating EPO, tumor-derived EPO, and pharmacologic ESA exposure because their spatial and temporal profiles differ. Third, it applies explicit operational evidence categories to each cellular compartment and explains why expression evidence, functional evidence, and causal in vivo evidence are not interchangeable. Fourth, it converts methodological concerns into a tiered, feasible research roadmap that accommodates routine human biopsy studies, as well as advanced mechanistic models.

The aim is not to portray EPO as uniformly protumorigenic or to dismiss informative exploratory studies. Instead, we identify what each study design can establish, summarize where EPO-related effects are reproducible or contradictory, and define proportionate next steps for clinical and laboratory research. This approach is intended to support cautious translation into cancer immunotherapy while preserving the legitimate supportive care role of ESAs.

## 2. Review Methods and Evidence Appraisal Framework

### 2.1. Literature Identification and Scope

This study is a critical narrative review rather than a systematic review or meta-analysis. Targeted PubMed searches were updated through August 2026 using combinations of the terms “erythropoietin,” “erythropoietin receptor,” “cancer,” “tumor,” “immunity,” “macrophage,” “dendritic cell,” “CD71 erythroid cell,” “angiogenesis,” “receptor detection,” and “erythropoiesis-stimulating agent.” The reference lists of relevant primary studies, reviews, meta-analyses, and clinical guidelines were also examined. Priority was given to studies that identified the EPO source, the responding cellular compartment, receptor dependence, exposure conditions, and an in vivo tumor or immune outcome. Cancer-specific evidence was interpreted separately from findings in transplantation, autoimmunity, renal disease, infection, or isolated cell systems.

### 2.2. Operational Evidence Categories

**Figure 2:**
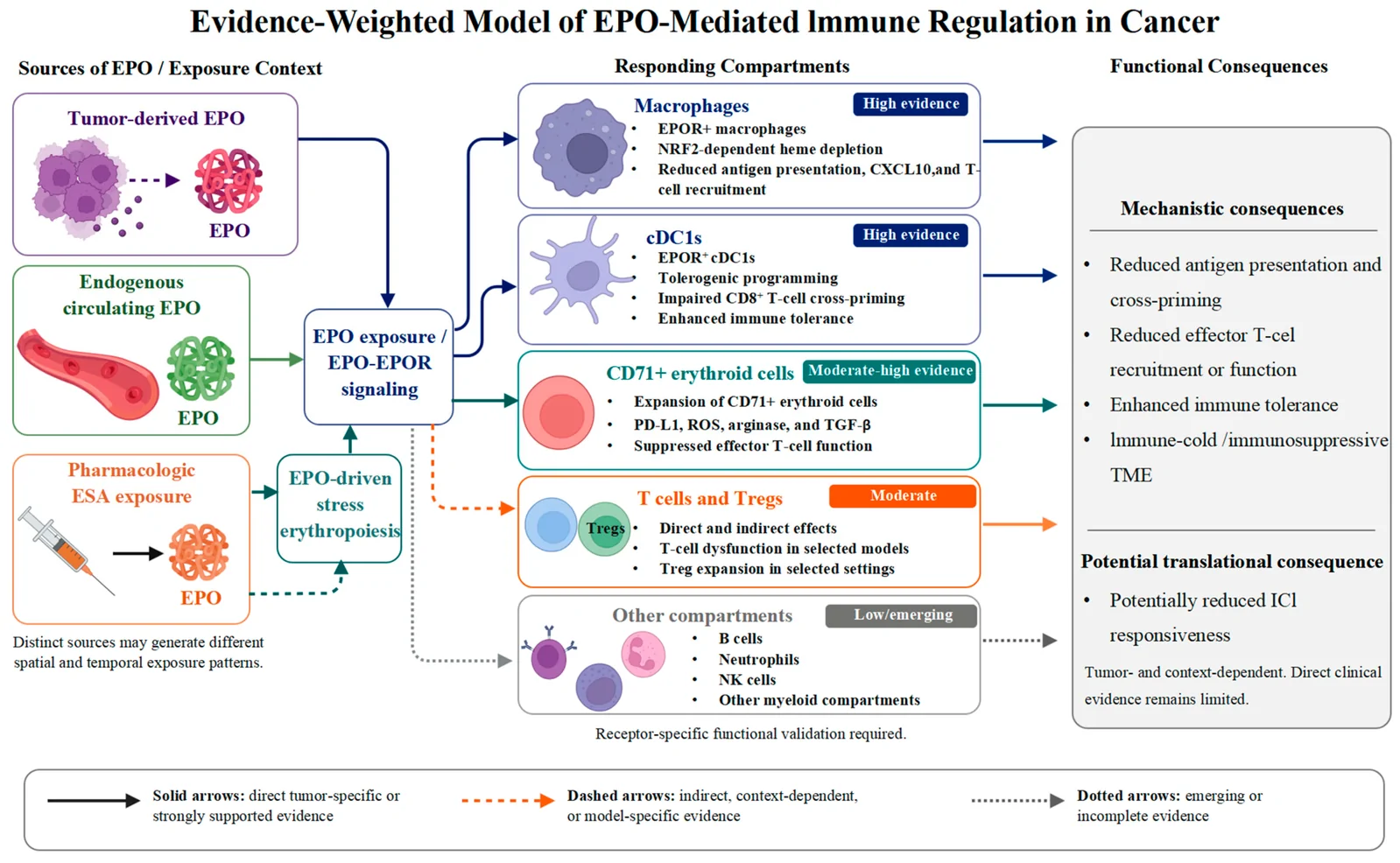
Evidence-weighted, integrated model of EPO-mediated immune regulation in cancer. Distinct EPO sources converge on EPOR-positive or EPO-responsive compartments. The effects of tumor-derived EPO on EPOR-positive macrophages are supported by direct mechanistic evidence from tumor models, and lineage-restricted EPOR deletion establishes a causal tolerogenic role for cDC1s. EPO-associated stress erythropoiesis expands CD71-positive nucleated erythroid cells that can suppress effector immunity. Effects on T cells, B cells, neutrophils, and other compartments are more context-dependent. The right-hand panel links impaired antigen presentation and T-cell function to an immune-cold microenvironment and identifies potential intervention points. Solid arrows denote direct tumor-specific or strongly supported evidence; dashed arrows denote indirect, model-specific, or context-dependent evidence; dotted arrows denote emerging evidence. AI assistance was used during the initial visual design of this conceptual schematic. The current figure was drawn by the authors using graphical software and was scientifically verified by the authors; it contains no primary experimental data. Abbreviations: cDC1, conventional type 1 dendritic cell; EPO, erythropoietin; EPOR, erythropoietin receptor; ESA, erythropoiesis-stimulating agent; ICI, immune checkpoint inhibitor; NRF2, nuclear factor erythroid 2-related factor 2; ROS, reactive oxygen species; TME, tumor microenvironment; Treg, regulatory T cell.

The evidence categories in Figure 2 and Table 1 are transparent narrative review categories and were not derived from a validated certainty-of-evidence instrument. They were applied at the level of a biological claim and cellular compartment rather than assigned solely based on publication count. “High” evidence requires direct relevance to a tumor model, receptor- or ligand-specific perturbation, and an in vivo functional outcome related to tumor biology or immunity; human corroboration increases confidence but is not mandatory when causal human experimentation is impossible. “Moderate-to-high” evidence combines complementary preclinical and human findings but lacks a complete receptor-specific causal chain. “Moderate” evidence includes reproducible functional observations with incomplete tumor specificity, receptor attribution, or in vivo confirmation. “Variable” denotes conflicting results across models or substantial dependence on assay validity or exposure. “Low/emerging” indicates that the evidence is derived mainly from non-tumor settings, isolated cells, or association without receptor-specific functional validation. These categories were refined during synthesis and were not prospectively registered; their purpose is to make interpretive judgments explicit and reproducible, not to imply formal grading.

### 2.3. Use of Generative Artificial Intelligence

OpenAI ChatGPT 5.6 (OpenAI, San Francisco, CA, USA; accessed between 4 August and 10 September 2026) was used as an editing aid for English-language refinement. AI assistance was used during the initial visual design of the conceptual schematics. The current versions of Figure 1 and Figure 2 were drawn by the authors using graphical software. All mechanistic content, arrows, evidence assignments, citations, and interpretations were checked against the cited literature and approved by the authors. No generative artificial intelligence tool was used to create, modify, analyze, or enhance primary experimental data, clinical images, blots, photographs, radiographs, measurements, or quantitative data visualizations. This review reports no newly generated experimental data. The authors take full responsibility for the accuracy and integrity of the manuscript and figures.

## 3. Biological Framework of EPO/EPOR Signaling

### 3.1. Oxygen Sensing, EPO Production, and Canonical Erythroid Signaling

EPO is a heavily glycosylated cytokine whose circulating half-life and biological activity are influenced by its carbohydrate structure. Under normoxia, hypoxia-inducible factor (HIF)-alpha proteins are hydroxylated and targeted for degradation through the von Hippel–Lindau pathway. Reduced oxygen tension stabilizes HIF-2α in renal EPO-producing cells, allowing transcriptional activation of the EPO gene and a coordinated increase in erythropoiesis [1,2,17]. The liver is the principal source of EPO during fetal development and can contribute under selected pathological conditions, including renal anemia [18]. In tumors, hypoxia, oncogenic signaling, inflammation, and tissue injury may create additional local sources of EPO, although their magnitude and cellular origin vary by tumor type.

EPOR is present as a preassembled or ligand-responsive homodimer on erythroid progenitors. Ligand binding changes receptor geometry, activates associated JAK2 molecules, and initiates phosphorylation of EPOR cytoplasmic motifs. STAT5 dimerization and nuclear translocation induce survival and differentiation programs, including BCL2L1 (Bcl-xL). Parallel PI3K/AKT and RAS/RAF/MEK/ERK pathways regulate metabolism, cell cycle progression, and stress resistance. Negative feedback is mediated by suppressor of cytokine signaling proteins, phosphatases, receptor internalization, ubiquitination, and proteasomal or lysosomal degradation [3,19,20,21]. This circuitry is established in erythroid cells; its operation in a malignant, immune, or stromal compartment should be demonstrated rather than inferred from expression alone.

### 3.2. EPOR Detection in Tumors: A Tiered Approach to Expression and Function

Reports of EPOR in solid tumors have been inconsistent. Several early studies relied on polyclonal antibodies, including C-20 and M-20, which detected proteins of incorrect molecular weight, cross-reacted with non-EPOR proteins, or produced staining in EPOR-negative controls [6,22]. More selective detection was achieved by developing the rabbit monoclonal antibody A82 against a defined human EPOR cytoplasmic epitope and validating it with EPOR-positive and EPOR-negative cell systems, immunoblotting at the expected molecular mass, immunoprecipitation, and quantitative comparisons with erythroid controls [23,24]. These studies found very low EPOR abundance in many tumor cell lines and did not detect EPOR proteins in several primary breast, lung, colorectal, ovarian, and skin tumor samples; functional signaling through cell-surface EPOR was likewise undetectable in an assessment of a broad panel of tumor cell lines [25].

Human biopsy material nevertheless remains valuable. The appropriate question is whether a study is screening for receptor localization or claiming receptor-mediated function. For exploratory screening, a pragmatic minimum is multiplex immunohistochemistry or immunofluorescence using a validated monoclonal antibody, a known EPOR-positive erythroid control, an EPOR-negative control, an isotype or secondary-only control, and cellular co-localization markers. Orthogonal RNA in situ hybridization or targeted transcript analysis can strengthen confidence in cellular identity. Such studies can map candidate EPOR-positive populations and generate hypotheses, but expression alone cannot establish ligand responsiveness. When fresh tissue is available, flow cytometry using a validated reagent targeting the extracellular domain of EPOR, ligand-binding assays, or targeted proteomics can provide stronger evidence of surface accessibility.

Intracellular EPOR requires separate interpretation. A newly synthesized receptor passes through the endoplasmic reticulum and Golgi, whereas a ligand-occupied surface EPOR is rapidly internalized and routed predominantly toward ubiquitin-dependent proteasomal and lysosomal processing [20,21]. Limited recycling has been observed under selected experimental conditions, but intracellular immunoreactivity may also reflect biosynthetic intermediates, retained or misfolded proteins, or internalized receptors. Canonical signaling begins at the plasma membrane where extracellular EPO can bind to EPOR; a sustained signaling role for an intracellular EPOR pool in tumor cells has not been established. Thus, absence of strong steady-state surface staining may reflect low abundance and rapid trafficking, but intracellular staining alone is not evidence of a functional EPO response.

Functional attribution should be scaled to the claim. A study of ligand responsiveness should demonstrate a dose- and time-dependent biochemical effect, include appropriate positive and negative controls, and, when technically feasible, show loss of the response after EPOR blockade, knockdown, or deletion. Rescue experiments and lineage-restricted genetics provide the strongest causal evidence but are not prerequisites for descriptive human tissue mapping. Conversely, a biopsy study should not be dismissed because genetic manipulation is impossible; its conclusions should be limited to validated localization and association. This two-stage framework preserves the clinical value of human tissues while preventing expression data from being overinterpreted as receptor-mediated biology.

EPOR should also be distinguished from the EPH receptor family. EPH receptors are receptor tyrosine kinases encoded by different genes. For example, EPO has been reported to promote tumor growth through EphB4 in selected models [26], but such findings do not demonstrate canonical EPOR signaling. Precise receptor nomenclature is therefore essential.

### 3.3. Endogenous, Tumor-Derived, and Pharmacologic EPO Are Distinct Exposures

Endogenous circulating EPO is an integrated response to hemoglobin concentration, renal function, oxygenation, inflammation, iron availability, bleeding, and marrow demand. Tumor-derived EPO may instead act locally within hypoxic niches and generate paracrine exposure that is not captured by a single serum measurement. Pharmacologic ESAs create a third pattern, with product- and route-dependent peaks, half-lives, and cumulative exposure. These categories should be analyzed separately because equal nominal concentrations do not imply equal duration, location, receptor occupancy, or biological effect.

A tumor may therefore display local EPO signaling despite a serum concentration within the laboratory reference interval, whereas severe anemia may produce high circulating EPO without a tumor-specific program. Similarly, an association between ESA use and outcome cannot identify whether the mediator is a malignant cell, an immune cell, the vasculature, thrombosis, correction of hypoxia, or confounding by anemia severity. Translational studies should record the source, concentration, timing, product, route, and responding compartment rather than treating “EPO exposure” as a single variable.

## 4. Tumor-Intrinsic and Vascular Effects

### 4.1. Survival, Proliferation, Stemness, and Treatment Resistance

The available evidence does not support a universal effect of EPO on tumor cells. Positive results have been reported in selected models with demonstrable receptor-dependent signaling. EPO activated JAK2/STAT5, PI3K/AKT, or ERK pathways, activated anti-apoptotic programs, or altered invasion in engineered breast cancer cells, EPOR-dependent breast cancer models, hepatocellular carcinoma, melanoma, and head and neck squamous cell carcinoma [27,28,29,30,31]. In prostate cancer models, EPO supported survival under stress without consistently increasing primary growth or bone metastasis, demonstrating that survival and progression are not equivalent endpoints [32].

Additional model-specific studies have implicated EPO/EPOR signaling in breast, ovarian, and renal tumor cell phenotypes [33,34,35,36]; stem-like cell maintenance or self-renewal in glioma and breast cancer [37,38]; and resistance-related phenotypes during chemotherapy or HER2-directed treatment [39,40,41]. These findings broaden the range of reported responses but do not obviate the need for receptor-specific validation in each model.

Negative studies are equally informative. Several tumor cell lines and primary tissues contain a low level of ligand-accessible EPOR, show no reproducible EPO-induced phosphorylation, or fail to increase proliferation after exposure [24,42]. Differences across reports arise not only from genuine tumor heterogeneity but also from antibody specificity, receptor abundance, engineered overexpression, ligand concentration, serum conditions, exposure duration, and contaminating erythroid or stromal cells. Alternative receptors, including EphB4 in specific models, further complicate interpretation [26].

The most defensible answer is therefore conditional: EPO can promote survival, invasion, or therapy resistance in molecularly defined models, but these effects are neither uniform across solid tumors nor predictable from EPOR mRNA or routine immunostaining alone. For each tumor type, the relevant questions are whether ligand-accessible receptor is present, whether EPO produces a receptor-dependent signal at a clinically interpretable exposure level, and whether that signal changes an in vivo tumor phenotype. This conclusion synthesizes the positive and negative literature without requiring every exploratory study to meet the highest standard of causal validation.

### 4.2. Angiogenesis and Vasculogenic Mimicry

EPO can promote endothelial survival, migration, and neovascularization in experimental systems [43,44,45,46]. In EPOR-negative tumor models, exogenous EPO accelerated growth through host-mediated angiogenesis, demonstrating that a vascular effect can occur without direct tumor cell signaling [47]. In hepatocellular carcinoma, the co-expression of EPO/EPOR and microvessel density has been associated with angiogenesis, whereas vasculogenic mimicry—tumor cell-lined channels that resemble vascular structures—has been linked to aggressive disease [48,49]. Evidence that EPO directly causes vasculogenic mimicry is weaker than evidence for angiogenic or hypoxia-adaptive effects, and the processes should not be conflated.

Additional model-specific observations linked EPO to glioma angiogenesis, lymph node lymphangiogenesis and metastasis, or postoperative recurrence in a murine colon cancer model through a vascular endothelial growth factor-independent mechanism [50,51,52]. These findings support vascular and stromal routes while remaining dependent on tumor type, exposure, and experimental context.

Abnormal vessels, high interstitial pressure, and persistent hypoxia can limit lymphocyte trafficking, favor suppressive myeloid populations, and reduce drug delivery. Conversely, correction of severe anemia can improve oxygen transport. Because these effects may oppose one another, angiogenesis markers alone cannot predict the net consequence of EPO. Studies should distinguish vessel density from perfusion and normalization, and they should evaluate oxygenation, immune cell localization, and tumor growth in the same model when feasible.

## 5. EPO as a Regulator of Tumor Immunity

### 5.1. Tumor-Associated Macrophages: The Strongest Tumor-Specific Evidence

Tumor-associated macrophages (TAMs) provide the clearest evidence that EPO functions as an immunoregulatory signal in cancer. In liver cancer models, tumor-derived EPO engaged EPOR on resident macrophages and activated an NRF2-dependent program associated with heme depletion. The resulting state reduced antigen presentation and CXCL10 production, limited T-cell recruitment, and produced a non-inflamed tumor microenvironment. Genetic depletion of tumor EPO or macrophage EPOR increased inflammatory features and improved tumor control, including responsiveness to immune checkpoint blockade [8,9]. This study identified the ligand source, responding cell, intracellular mechanism, and therapeutic consequence within one causal framework.

Earlier work also showed that macrophages can respond to EPO, although the direction of the response varied with the model and context. EPO enhanced pro-inflammatory functions in murine macrophage systems, whereas other studies linked macrophage EPO signaling to dying cell clearance, inflammation resolution, immune tolerance, or macrophage-mediated T-cell suppression [53,54,55,56]. These apparently divergent findings reinforce the importance of tissue context, differentiation state, dose, and co-stimulation. They also explain why the current tumor-derived EPO–NRF2/heme program should not be reduced to a generic M1/M2 label.

Future studies should map EPOR-positive macrophages across tumor types; determine whether they arise from resident or recruited populations; and establish whether anemia, iron restriction, or ESA exposure modifies their abundance or state. Human biopsy studies can begin with validated multiplex localization and combined assessment of heme status and antigen presentation; cell-specific knockout is a gold standard for causal animal studies, not a universal entry requirement.

### 5.2. Conventional Type 1 Dendritic Cells and Immune Tolerance

A recent study extended EPO immunobiology to cDC1s, a dendritic cell subset specialized for cross-presentation and priming of tumor-reactive CD8-positive T cells. Cell-specific deletion of EPOR in cDC1s reduced tumor growth, enhanced antitumor T-cell immunity, increased precursor-exhausted tumor antigen-specific CD8-positive T cells, and reduced intratumoral regulatory T cells [10]. These results establish a lineage-specific role for EPOR in regulating the threshold for immune tolerance in a second antigen-presenting cell compartment.

Earlier non-tumor studies reported that EPO can alter dendritic cell survival, maturation, co-stimulatory molecules, and cytokine secretion [57,58]. The contrast between these immunostimulatory observations and tumor-associated cDC1 tolerance again argues against extrapolating across cell states and disease contexts. In cancer, the cDC1 study carries greater evidentiary weight because it links receptor deletion to antigen-specific immunity and tumor control in vivo.

These findings support cell-selective EPO/EPOR interruption but do not justify systemic blockade. Broad inhibition could impair erythropoiesis, whereas indiscriminate dendritic cell activation could increase inflammatory toxicity. The translational objective is therefore to identify tumor-localized delivery systems, receptor interfaces, or downstream modules that separate immune tolerance from erythroid survival.

### 5.3. CD71-Positive Nucleated Erythroid Cells as Systemic and Intratumoral Suppressors

Cancer-associated stress erythropoiesis can mobilize immature CD71-positive erythroid cells, including nucleated erythroid progenitors, into blood, the spleen, and tumors. The term “CD71-positive nucleated erythroid cells” is used here when the reported population retains a nucleus; phenotypic definitions should also include erythroid-lineage markers because CD71 alone is not lineage-specific. Foundational studies showed that immature CD71-positive erythroid cells can suppress immunity through arginase, and tumor studies identified PD-L1, reactive oxygen species, transforming growth factor-β, myeloid differentiation, and checkpoint interactions as additional mechanisms [11,12,13,59].

In patients with virus-associated solid tumors, increased CD71-positive erythroid cells were associated with impaired effector T-cell function and unfavorable immunotherapy outcomes [12]. Because EPO is a principal driver of erythroid expansion, this compartment provides a plausible link between systemic anemia biology and tumor immunity, even when malignant cells lack functional EPOR. However, the links to EPO exposure and direct EPOR dependence in these cells are less fully established than the corresponding mechanisms in macrophages and cDC1s. The evidence is therefore rated moderate-to-high rather than high.

Before these cells are used as biomarkers, studies should report nucleation status, gating strategy, erythroid markers, hemoglobin, reticulocyte count, iron indices, renal function, inflammation, bleeding, transfusion history, and ESA exposure. Longitudinal sampling is particularly important because stress erythropoiesis can change rapidly during treatment.

### 5.4. T Cells and Regulatory T Cells: Direct and Indirect Effects

EPO can influence T-cell biology, but the direction and clinical meaning are context-dependent. In transplantation and autoimmune disease settings, EPO reduced T follicular helper cell differentiation, blunted antibody responses, and expanded regulatory T cells [14,15]. These observations demonstrate immune modulation but do not prove that systemic EPO directly suppresses tumor-reactive T cells in patients with cancer. Reports of EPOR on circulating lymphocytes also remain method-dependent [60]. In tumors, many apparent T-cell effects may be secondary to altered antigen presentation, macrophage chemokine production, cDC1 tolerance, or expansion of CD71-positive nucleated erythroid cells.

A preclinical triple-negative breast cancer study found that EPO promoted tumor progression and increased CD39 expression on immune cells, consistent with a tolerogenic purinergic environment [16]. This finding supports a link between pharmacologic EPO and immune suppression in that model but should not be generalized across tumor types or dosing regimens. Descriptions of T-cell effects should distinguish direct from indirect mechanisms according to the study design, and claims of direct effects require cell-specific evidence of receptor involvement.

### 5.5. B Cells, Neutrophils, and Other Compartments: Emerging Evidence

Studies in non-cancer settings have reported changes in B-cell distribution, antibody production, neutrophil survival, reactive oxygen species, natural killer cell activity, and myeloid-derived suppressor populations after EPO exposure [53,57,60]. These findings are biologically informative but are derived mainly from inflammation, infection, transplantation, renal disease, or high-dose in vitro systems. They should therefore be treated as hypothesis-generating for tumor immunity rather than as established components of a universal EPO network.

A feasible next step is to prioritize compartments identified using spatial human data or reproducible functional screening and then apply receptor blockade or genetic perturbation in the most informative models. Negative results help define which cellular compartments are responsive and prevent unsupported extension of the proposed signaling network.

## 6. Evidence Map and Pragmatic Methodological Priorities

The literature is most interpretable when the biological compartment, EPO source, exposure pattern, model type, and level of causal validation are considered together. Table 1 states both the operational evidence level and the reason for each assignment.

### Minimum, Enhanced, and Gold-Standard Designs

Experimental rigor should be proportional to the study objective. Table 2 provides workable designs for human tissue, cell systems, animal models, and clinical cohorts. The minimum level is intended to generate interpretable evidence in laboratories with standard resources; enhanced and gold-standard elements provide greater causal resolution when feasible.

“Physiological” and “pharmacological” exposure should be defined operationally rather than by a universal concentration cutoff. Physiological exposure refers to the distribution of endogenous EPO concentrations measured in untreated participants or animals matched for species, anemia severity, renal function, oxygenation, and assay method. Pharmacological exposure refers to the concentration–time profile after administration of a specified ESA using a defined dose, route, and schedule. In vitro studies should bracket disease-relevant endogenous concentrations and clinically observed post-dose exposure when such data are available; they should not rely on a single high concentration. Because epoetin alfa and darbepoetin alfa have distinct pharmacokinetics, studies should report baseline concentration, dose, route, time since dose, peak concentration (Cmax), time to peak (Tmax), area under the concentration–time curve (AUC), trough concentration, and terminal half-life when relevant [61,62].

The observation period should match the biological endpoint. Proximal phosphorylation is usually assessed over minutes to a few hours, transcriptional changes over several hours, immune phenotype and viability over approximately 24–72 h, and tumor or hematologic outcomes over a justified longitudinal interval. These ranges are not substitutes for pilot data. Studies should also report ESA lot, formulation, purity, endotoxin control, medium conditions, species compatibility, and whether concentrations are expressed in activity units or molar terms.

## 7. Clinical Context: ESAs in Patients with Cancer

### 7.1. Historical Survival and Thromboembolic Concerns

Clinical concern about EPO in cancer was driven by randomized trials in which ESAs were used outside contemporary indications or to pursue high hemoglobin targets. In head and neck cancer, epoetin beta given during curative radiotherapy was associated with worse locoregional control and survival. In metastatic breast cancer, an epoetin alfa strategy targeting higher hemoglobin was associated with decreased survival [63,64]. Meta-analyses subsequently identified increased thromboembolic risk and raised concern about mortality in some settings [65,66,67]. These findings established a durable safety principle: correction of anemia is not biologically neutral, and supportive care benefit must be balanced against vascular and oncological risks.

Other randomized studies conducted with contemporary chemotherapy regimens reported heterogeneous disease control and hematologic outcomes, reinforcing the need to interpret ESA effects according to treatment intent, hemoglobin target, regimen, exposure, and thromboembolic risk rather than assuming a uniform class effect across settings [68,69,70].

The trials do not establish that the adverse outcomes were caused by direct EPOR signaling in malignant cells. Potential explanations include thrombosis, changes in blood viscosity, endothelial effects, altered tumor oxygenation, immune modulation, and differences in treatment dose intensity. In observational studies, associations between ESA use and outcomes may additionally be affected by confounding related to anemia severity. Nevertheless, the trials are clinically decisive because they define practices that should not be repeated: ESA use without an accepted indication, treatment in settings in which cure is intended without compelling justification, and dosing to normalize hemoglobin.

### 7.2. Current Clinical Positioning

The 2019 ASCO/ASH guideline supports ESA use in selected patients with chemotherapy-associated anemia when cancer treatment is not intended to be curative and hemoglobin has fallen below 10 g/dL; red blood cell transfusion remains an alternative [71]. ESAs should generally not be offered for most cases of non-chemotherapy-associated anemia, and the lowest dose sufficient to reduce transfusion requirements should be used. Iron status and reversible causes of anemia should be evaluated before and during therapy. These positions are consistent with broader meta-analytic and European guidance [72,73,74].

Emerging immune mechanisms do not justify withholding indicated ESA therapy from every patient, nor do they support administering EPO as an immunotherapy. They do, however, support more detailed prospective documentation of ESA exposure and its clinical context. Immunotherapy trials and real-world cohorts should record the ESA product, dose, route, timing, hemoglobin trajectory, iron therapy, transfusions, renal function, inflammatory markers, and thromboembolic events.

### 7.3. EPO and Immune Checkpoint Therapy

Direct clinical evidence showing that ESA exposure influences immune checkpoint inhibitor efficacy remains limited. Macrophage, cDC1, and CD71-positive nucleated erythroid cell studies provide evidence of biological plausibility but do not define the magnitude, direction, or tumor-type dependence of a clinical interaction. Retrospective analyses should avoid immortal time bias and confounding by indication because ESA recipients may have more severe anemia, longer treatment exposure, renal dysfunction, or advanced disease.

Practical prospective studies could collect baseline and serial blood samples before and after ESA initiation, with optional paired tumor samples when clinically available. Immune cell states and outcomes could then be compared between patients receiving ESAs and those managed with a clearly defined transfusion or iron therapy strategy. These should initially be safety and biomarker studies, not trials of EPO as anticancer treatment.

## 8. Translational Opportunities

### 8.1. Biomarkers: A Two-Tier Clinical and Research Strategy

Serum EPO should not be used as a standalone oncological biomarker. Its concentration is affected by hemoglobin, renal function, oxygenation, inflammation, iron availability, blood loss, transfusion, ESA exposure, assay platform, and sampling time. Patients with cancer may also show an EPO response that is inadequate for the degree of anemia [75]. A practical first-tier panel should therefore include serum EPO, a complete blood count, reticulocyte count, ferritin, transferrin saturation, C-reactive protein, and creatinine or estimated glomerular filtration rate, interpreted alongside information on recent bleeding, transfusion history, and ESA product, dose, and timing. This panel is inexpensive, clinically interpretable, and suitable for large cohorts.

A second-tier research panel can be reserved for mechanistic studies or biomarker enrichment. It may include tumor EPO expression or local protein concentration, EPOR-positive macrophage or cDC1 signatures, heme/NRF2 and antigen presentation markers, CD71-positive nucleated erythroid cell abundance and suppressive function, and spatial exclusion of effector T cells. These measures are intended for research rather than routine bedside testing. Their immediate role is to identify which element of the source–responder–effect axis adds information beyond the routine panel. Only components that are analytically reproducible, affordable, and independently validated should progress toward clinical use.

Biomarker development should proceed through analytic, biological, and clinical validation. A useful marker must distinguish EPO pathway activity from general illness and demonstrate added value beyond routine covariates. Prespecified cutoffs, external validation, and decision curve or incremental performance analyses are necessary before clinical implementation.

### 8.2. Therapeutic Blockade: Existing Prototypes and Remaining Constraints

Macrophage and cDC1 studies suggest that interruption of EPO/EPOR signaling could convert selected immune-cold tumors into more inflamed, checkpoint-responsive states. No selective EPO/EPOR-directed anticancer therapy is currently approved. Systemic EPO neutralization or broad EPOR blockade would be expected to impair erythropoiesis and could worsen anemia, tissue hypoxia, fatigue, and treatment tolerance. JAK inhibitors are not selective for EPOR and should not be considered validated EPO-targeting agents in solid tumors.

Several early preclinical prototypes demonstrate feasibility but not clinical readiness. In mammary tumor window chamber and fat pad models, locally administered recombinant soluble EPOR, a neutralizing anti-EPO monoclonal antibody, and tumor cell expression of a secreted antagonist (R103A-EPO) reduced angiogenesis or delayed early tumor growth [76]. The peptide antagonist EMP9 has shown antiproliferative and pro-apoptotic effects in experimental cancer systems, but the supporting evidence is limited and has not established a therapeutic window in patients [77]. These examples should be viewed as proof of concept. We found no established modern program demonstrating selective intratumoral delivery of an EPO pathway inhibitor together with preserved marrow erythropoiesis.

Future strategies may include tumor-targeted ligand traps, macrophage- or dendritic cell-directed nanoparticles, bispecific localization, or inhibition of immune-specific downstream programs such as the macrophage NRF2/heme axis. Local delivery is attractive because it may reduce systemic hematologic toxicity, but biodistribution, escape into the circulation, immunogenicity, and intratumoral target engagement must be measured. Combination with immune checkpoint blockade should begin only after exposure–response and hematologic stopping rules are defined.

### 8.3. Non-Erythropoietic Derivatives: Mechanism-Tailored Safety Testing

Non-erythropoietic EPO derivatives and alternative receptor models emerged from tissue injury research [78,79,80], but their receptor biology and relevance to cancer immunity remain incompletely resolved. Preclinical evaluation should be tailored to the candidate’s proposed mechanism of action. All candidates should undergo a core assessment of molecular identity, intended receptor engagement, canonical EPOR activation, and erythroid colony formation at clinically relevant exposures. For a compound designed to avoid canonical EPOR activation, the absence of such activation should be confirmed experimentally; however, testing need not follow the same framework used for a full agonist.

Additional testing should be guided by the proposed mechanism of action and biodistribution. A macrophage-targeted agent requires macrophage phenotype and function assays; a dendritic cell-directed agent requires antigen presentation and T-cell-priming assays; a systemically distributed agent requires broader tumor cell, angiogenesis, thrombosis, marrow, and tissue repair evaluation. Findings from the core screen should determine which modules are expanded. This staged approach protects patients without making early development unnecessarily prohibitive.

## 9. Prioritized Research Agenda

The research agenda is organized into three levels. The minimum acceptable level emphasizes validated reagents, clearly defined exposure conditions, feasible localization methods suitable for human tissues, and at least one proximal signaling readout and one functional readout. The enhanced level adds orthogonal detection, perturbation, longitudinal sampling, pharmacokinetics, and matched anemia controls. The gold-standard level integrates spatial or single-cell atlases, lineage-restricted genetics, compartment-selective interventions, and clinically meaningful safety and efficacy endpoints. Not every laboratory must perform every level; smaller studies can make rigorous contributions when their claims remain proportionate to their design.

## 10. Conclusions

EPO is not adequately described as an erythroid hormone with occasional off-target effects. It is a context-dependent signal connecting hypoxia, stress erythropoiesis, antigen-presenting cells, vascular biology, and adaptive immunity. The strongest causal tumor evidence currently involves EPOR-positive macrophages and cDC1s. CD71-positive nucleated erythroid cells provide an additional systemic and intratumoral route to immune suppression. Direct tumor cell signaling occurs in selected models but is not a universal property of solid tumors.

Validated screening of human tissues can identify candidate cell populations, and orthogonal assays and perturbation studies can then test their functional relevance in suitable experimental models. For clinical practice, ESAs remain restricted supportive care drugs and are not immunotherapies. The main translational opportunity is to identify tumors in which a defined EPO source engages a specific suppressive compartment and to interrupt that interaction without worsening anemia. Practical biomarker panels, proportionate validation, and safety-preserving drug design will determine whether the EPO/EPOR axis becomes clinically useful.

## Figures and Tables

**Table 1:** Evidence hierarchy for EPO/EPOR effects in cancer. Ratings are narrative operational categories based on tumor specificity, receptor- or ligand-specific perturbation, in vivo functional causality, and human corroboration; they are not formal certainty-of-evidence grades. A study need not use every advanced method to contribute valid evidence. Rather, the strength of the conclusion should match the design. other abbreviations are defined in the text.

Domain	Representative Evidence	Evidence Level	Interpretation	Principal Limitation
Tumor-associated macrophages	Tumor-derived EPO; macrophage EPOR; NRF2/heme reprogramming; genetic perturbation; tumor and ICI outcomes	High	Complete source–responder–mechanism–outcome chain in tumor models	Breadth across human tumor types remains unknown
cDC1s	Lineage-specific EPOR deletion; antigen-specific CD8-positive T-cell and tumor outcomes	High	Direct causal tumor evidence for a tolerogenic EPOR function	Independent models and human validation are limited
CD71-positive nucleated erythroid cells	Human association plus functional immune suppression in tumor and developmental settings	Moderate-to-high	Plausible systemic and intratumoral suppressive route	The EPO/EPOR-specific causal chain remains incomplete
T cells and regulatory T cells	Direct and indirect effects across transplantation, autoimmunity, and selected tumors	Moderate	Context-dependent immune modulation is biologically credible	Direct receptor dependence in tumor-reactive T cells is often unresolved
Tumor cells	Positive receptor-dependent models contrasted with low-receptor and negative response models	Variable	Survival, invasion, or resistance can occur in molecularly defined contexts	Assay validity, engineered overexpression, dose, and tumor heterogeneity
Endothelium and angiogenesis	Host-mediated angiogenesis and vascular associations	Moderate	Vascular effects can occur without tumor cell EPOR	Perfusion, normalization, and direct EPO causality are not consistently separated
B cells, neutrophils, NK cells, and other myeloid cells	Mainly non-tumor or isolated cell observations	Low/emerging	Provides hypotheses for compartment-specific tumor studies	Limited tumor-specific, receptor-dependent functional evidence
Clinical ESA exposure	Randomized trials, meta-analyses, and supportive care guidelines	High for safety and use	Defines clinical restrictions and thromboembolic risk	Does not isolate the immune or receptor-level mechanism

**Table 2:** Pragmatic study design matrix for EPO/EPOR research. Suggested time windows are starting points rather than universal mandates and should be justified for the model and ESA product. PK, pharmacokinetic; PD, pharmacodynamic.

Study Context	Minimum Feasible Design	Enhanced/Gold-Standard Options	Essential Reporting and Timing
**Human biopsy mapping**	Validated monoclonal EPOR reagent; known erythroid positive control; EPOR-negative and isotype/secondary-only controls; cellular co-localization	Orthogonal RNA in situ hybridization; fresh tissue flow cytometry; ligand binding or targeted proteomics; spatial profiling	Fixation, ischemia, and processing intervals; antibody clone/lot; scoring; cell identities; anemia, renal, inflammatory, transfusion, and ESA context
**Cell-based ligand response**	Vehicle, responsive positive control and receptor-negative control; named EPO/ESA product across an exposure range; proximal and functional readouts	EPOR blockade or knockdown; deletion and rescue when feasible; orthogonal surface quantification; unbiased transcriptomic or proteomic readout	Minutes to a few hours for phosphorylation; several hours for transcription; approximately 24–72 h for phenotype/viability; dose, units, lot, purity, endotoxin, medium, and species compatibility
**Animal tumor study**	Vehicle, exposure-only, inhibitor-only, and rational combination groups; baseline and serial hematology; justified tumor and immune endpoints	Anemia-matched controls; lineage-restricted genetics; rescue; biodistribution and tumor target engagement; exposure–response modeling	Product, dose, route, schedule, randomization/blinding; hemoglobin, reticulocytes, and iron indices; tumor sampling relative to dosing; thrombosis and organ toxicity
**Clinical or real-world study**	Precisely defined time zero; baseline and serial routine biomarker panel; product/dose/route/timing; clinically appropriate comparator; prespecified outcomes	Paired tissue when available; mechanistic immune panel; PK/PD substudy; propensity or target trial design; external validation	Hemoglobin trajectory, renal function, inflammation, iron therapy, transfusions, bleeding, thromboembolism, disease status, treatment intent, immortal time bias, and confounding by indication

## Data Availability

No new data were created or analyzed in this review. Data sharing is not applicable to this article.

## Abbreviations

AUC, area under the concentration–time curve; cDC1, conventional type 1 dendritic cell; Cmax, peak concentration; EPO, erythropoietin; EPOR, erythropoietin receptor; ESA, erythropoiesis-stimulating agent; HIF, hypoxia-inducible factor; ICI, immune checkpoint inhibitor; JAK2, Janus kinase 2; MAPK, mitogen-activated protein kinase; NRF2, nuclear factor erythroid 2-related factor 2; PD, pharmacodynamic; PI3K, phosphoinositide 3-kinase; PK, pharmacokinetic; ROS, reactive oxygen species; STAT5, signal transducer and activator of transcription 5; TAM, tumor-associated macrophage; Tfh, T follicular helper cell; Tmax, time to peak concentration; TME, tumor microenvironment; Treg, regulatory T cell; TSAT, transferrin saturation; VM, vasculogenic mimicry.
